# Comparison of ELISA and Three Rapid HCG Dipsticks in Diagnosis of Premature Rupture of Membranes

**Published:** 2011-06-01

**Authors:** N Kariman, M Hedayati, Z Taheri, M Fallahian, S Salehpoor, S H Alavi Majd

**Affiliations:** 1 Research Institute for Reproductive Health, Faculty of Nursing and Midwifery, Shahid Beheshti University of Medical Sciences, Tehran, Iran; 2 Obesity Research Center, Research Institute for Endocrine Sciences, Shahid Beheshti University of Medical Sciences, Tehran, Iran; 3 Ayatollah Taleghani Hospital, Shahid Beheshti University of Medical Sciences, Tehran, Iran; 4 Faculty of Paramedical Sciences, Shahid Beheshti University of Medical Sciences, Tehran, Iran

**Keywords:** Premature rupture of membranes, Human chorionic gonadotropin, Cervicovaginal discharge, Rapid test strip, ELISA

## Abstract

**Background:**

The importance of accurate diagnosis of premature rupture of membranes (PROM) is quite apparent while trying to diminish false negative or positive results as much as possible. This study compares Enzyme-Linked Immunosorbent Assay (ELISA) and three rapid human chorionic gonadothropin (HCG) dipsticks in diagnosis of premature rupture of membranes.

**Methods:**

During 2008-2009, 181 pregnant women with single pregnancy from 14 to 41 weeks of gestation who referred to Ayatollah Taleghani Hospital in Tehran, Iran were divided into two groups, 91 patients with PROM and 90 controls with matched gestational weeks. All patients underwent speculum examination for cervicovaginal washing fluid, HCG three rapid tests and ELISA.

**Results:**

The HCG concentration of vaginal fluid was significantly different between the two groups. Using receiver operating characteristic (ROC) curve and determining the threshold as 19 mIU/mL for HCG by ELISA method, the sensitivity was 94.5%; specificity, 91%; positive predictive value, 91.5%; negative predicted value, 94.2% and accuracy was 92.2%. In rapid diagnostic test, the most sensitivity was for ACON and the most specificity for DIMA. Comparing the four methods, DIMA strip showed the highest accuracy and the highest value in early diagnosis of ROM.

**Conclusion:**

The reliability of three rapid diagnostic tests in diagnosis of ROM in cervicovaginal discharge was acceptable.

## Introduction

Premature rupture of membranes (PROM) is defined as rupture of chorionic membranes before the beginning of labor at any time during pregnancy. [1][2] PROM occurs in 8-19.53% of term pregnancies[3][4] and 2-25% of all pregnancies.[5][6] PROM may lead to infection, cord prolapse, preterm and prolonged labor,[7][8] neonatal septicemia, respiratory distress syndrome9 and perinatal mortality.[3] The diagnosis of PROM is carried out by patient history and physical findings such as discharge of fluid in speculum examination, ferning pattern in microscopic exam, and pH metery of vaginal discharge with nitrazine test.[1][10] The diagnosis of PROM is easy in the presence of obvious rupture of membranes, while there are many false positive and negative results in the suspected cases that may lead to inappropriate interventions such as hospitalization and induction of labor. On the other hand, misdiagnosis of PROM may deprive the patient from appropriate treatments. Patient’s history is reliable only in 10-50% of cases.[10][11] Although, inspection of fluid leakage from cervix has been traditionally the only method for confirmed diagnosis of PROM, this method is associated with 12-30% false negative results.[12] Moreover, Nitrazine test may also lead to false positive or negative results. Also, Fern test has 13-30% false negative and 5-30% false positive results.[10][13]

Several studies have been conducted to find a definite, easy, non-invasive and reliable diagnostic test for PROM in recent years. These studies have mainly focused on biochemical agents with high concentration in amniotic fluid. These factors are prolactin, alfa feto protein (AFP), insulin-like growth factor, fibronectin and diamine oxidase.[6] However, the use of these tests, have not been associated with a considerable success rate.[14] Recently, human chorionic gonadotropin (HCG) in cervicovaginal discharges has been studied. Enzyme-Linked Immunosorbent Assay (ELISA) method for assessing beta subunit human chorionic gonadotropin (ßhCG) in cervicovaginal discharges in some studies has shown different diagnostic values.[6][10][14][15] HCG is a glycoprotein which is secreted by chorionic cytotrophoblast and exists in different amounts in mother’s serum and urine, as well as in amniotic fluid.8 Cooper et al.,[8] and Kariman et al.[16] were the only investigators who used urine one step ßhCG dipstick with cut-off value of 25 mIU/ml for diagnosis of PROM.

One step ßhCG dipstick is a diagnostic test for pregnancy which is easy, cheap, rapid and noninvasive, and midwives can easily use this method in all outpatient and inpatient settings. Furthermore, quantitative assay of HCG in cervicovaginal discharges is cheaper and more accessible than ßhCG kits. Thus, we conducted this study in order to compare the diagnostic value of three rapid ßHCG dipsticks and HCG ELISA in the cervicovaginal secretions for diagnosis of PROM.

## Materials and Methods

During 2008-2009, 181 pregnant women including 91 with PROM and 90 with intact membranes (controls) who referred to Prenatal Clinic or Emergency Department of Taleghani Hospital in Tehran, Iran with complaints of fluid discharge were enrolled. The information were collected by a data form including demographic data, results of speculum examination, fern and nitrazine test, HCG dipsticks (Acon Inc. from USA), and ELISA (dbc HCG ELISA, Diagnostics Biochem Co., Canada) results with accuracy of 99% and cut-off value of 25 mIU/ml; Cortez (CORTEZ DIAGNO STIC Co.,USA); and DIMA (DIMA Gesellschaft Fur Diagnostika Co., Germany) with cut-off value of 20 mIU/ml. The validity of data was confirmed by content validity method. Control solutions were utilized for confirming the validity of HCG dipsticks, and ELISA method. The reliability of data form was confirmed by test retest method. Moreover, the reliabilities of check list and speculum physical exam were established by Inter rater consistency.

Sampling was also performed by non-probability (convenience) method and sample size was determined based on the prevalence of PROM (5%), α=0.05 and ε=0.2. This study was approved by Ethics Committee of Shaheed Beheshti University of Medical Sciences. All patients were pregnant with singleton pregnancy and 20 to 41 weeks gestational age. Gestational age was determined based on the first day of last menstruation period in reliable cases, or one ultrasound in less than 14 weeks or two ultrasound documents among 14 to 24 weeks of pregnancy. The patients who were excluded were those with fetal anomalies, intrauterine fetal death, known disease, pregnancy complications, visible blood in vaginal secretions, use of vaginal drugs or intercourse in the prior night, meconium in amniotic fluid, and the patients with uterine contractions. Sampling of all patients was carried out by the same midwife and all laboratory tests were performed according the kits insert. After taking a written consent, pregnant women were examined in lithotomy position and leakage of fluid was inspected by sterile speculum and results were registered as positive, negative or suspicious. A cotton tip applicator was inserted in deep vagina and was immediately transferred on nitrazine paper; pH above 6.5 was considered positive. A sample of cervicovaginal secretion was taken in a similar method and was expanded on slides. The slide field was examined by microscope (10 magnifications) for diagnosis of ferning pattern. Five ml of sterile normal saline was injected in posterior fornix of vagina and then it was aspirated by the same syringe. Subsequently, three ml of this fluid was infused in a plastic tube and after five minute centrifuge, was transported to a microtube and maintained at -70°Ċ for next ELISA examination. Cut off value was determined by receiver operating characteristic curve (ROC). HCG dipsticks were immersed up to marked line in two ml of remaining fluid for ten to fifteen seconds, and the result of each test was read in 3 to ten minutes. The patients were taken as confirmed PROM group if they had (+) pooling and (+) nitrazine and (+) fern test results. Pregnant women with (-) pooling and (-) nitrazine and (-) fern test results were taken as control group. Statistical analysis was performed by SPSS software (version 16, Chicago, IL, USA); results were expressed as frequency, mean and standard deviation. Chi Square, student’s and Mann-withney tests were used for comparison of groups. Moreover, p value less than 0.05 was considered as statistically significant.

## Results

Demographic features of pregnant women with PROM and the control group are presented in Table 1.

**Table 1:** Descriptive data of the groups

**Groups**	**Control(Intact)**	**PROM**	**Statistical tests**	***P *value**
**variables**				
Age (year)	25.38±5.93	26.04±4.9	student’s-test	0.42
Education (school, high School)	44%	40.7%	Mann-withney	0.27
Job (household)	88.9%	92.3%	Chi^2^	0.47
Gestational age (week)	37.85±3.45	38.17±2.63	student’s-test	0.37
Gravida	1.73±1.06	1.76±1.24	Mann-withney	0.10
Parity	0.53±1.76	0.59±1.76	Mann-withney	0.40

**Fig. 1:**
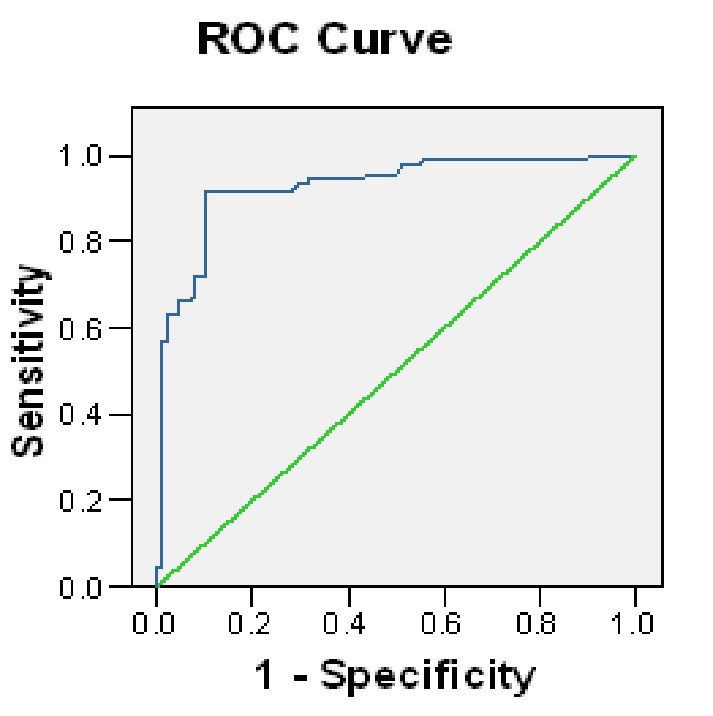
Receiving operator characteristic curve for vaginal HCG level

There was no statistically significant difference between the two groups. The mean HCG level in cervicovaginal discharge of PROM group was significantly higher than the control group [330.88±436.18 (1-2216) vs. 6.56±5.70 (1-769.70) mIU/mL; p=0.0001]. The cutoff value was 19 mIU/mL (Figure 1) which was determined by ROC for HCG in cervicovaginal discharge. With this cutoff value, the diagnostic value of parameters for HCG level in cervicovaginal discharge by ELISA were as follows: Sensitivity= 94.5%, specificity=91%, positive predictive value (PPV)=91.5%, negative predictive value (NPV)=94.2%, and accuracy=92.2%. This test had 8% false positive and 5% false negative results. Its positive likelihood ratio (PLR) and negative likelihood ratio (NLR) were 10.5% and 0.06%, respectively.

The result of 70 samples of the PROM group was positive and the result for 88 samples of the control group was negative with DIMA rapid test and a cutoff value of 20 mIU/mL. This test had 2% false positive and 23% false negative results. It’s PLR and NLR were 33.86% and 0.226%, respectively. The one step HCG ACON dipstick with cutoff value of 25 mIU/mL had 7% false positive and 9.8% false negative results. Its PLR and NLR were 11.25% and 0.06%, respectively. Furthermore, CORTEZ rapid HCG dipstick with cutoff value of 20 mIU/mL (78 samples of PROM group was positive and 88 of control group was negative) had 3% false positive and 14% false negative results. It’s PLR and NLR were 25.2% and 0.034%, respectively. The comparison of diagnostic values of the four methods is presented in Table 2.

**Table 2:** Diagnostic value of the four methods

**Diagnostic Power**	**Sensitivity(%)**	**Specificity(%)**	**PPV(%)**	**NPV(%)**	**Accuracy(%)**
**Methods**					
ELISA	94.5	91	91.5	94.2	92.2
ACON	90	92	92.1	90	91
DIMA	77.9	97.7	97.2	80.7	87.2
CORTEZ	85.7	96.6	96.2	87	91.1

## Discussion

This study showed that diagnostic powers of both three rapid HCG dipsticks and ELISA for PROM were in an acceptable range. The diagnostic power of a test is considered acceptable if all diagnostic parameters (e.g. Sensitivity, specificity, PPV and NPV) are more than 80%.[6] This study compared the diagnostic values of three rapid HCG dipsticks (ACON, DIMA, and CORTEZ) and ELISA on cervicovaginal discharge for diagnosis of PROM. DIMA dipstick had the highest PLR (33.86) and ELISA the lowest NLR (0.06); so DIMA dipstick was the most powerful test to confirm and ELISA the most powerful test for ruling out of PROM.

By comparing the three methods, one step dipstick revealed that ACON had the highest sensitivity and DIMA the highest specificity, PPV, NPV and the lowest false positive results. However, DIMA had the lowest sensitivity among the three HCG dipsticks. Overall, all diagnostic parameters of ACON dipstick were more than 90%. CORTESZ dipstick had the highest specificity and PPV (>96%) and an acceptable sensitivity and predictive values (>85%).

In Kariman et al.'s study (2008), the sensitivity of ACON dipstick for diagnosis of PROM was 97.7%, and its specificity, PPV, NPV, and accuracy were 88.4%, 89.4%, 97.5%, and 93%, respectively.[6] Cooper et al. (2004) reported that sensitivity, specificity, PPV, and NPV of one step ßhCG dipstick (cutoff= 25 mIU/mL) for diagnosis of PROM were 79%, 96%, 95%, and 84%, respectively.[8] Our results are in agreement with these two studies. These dipsticks are now available for diagnosis of pregnancy in urine specimen. During pregnancy, the amount of HCG in amniotic fluid varies from 2000 to 70000 mIU/mL.[10] After rupture of fetal membranes, HCG can be detectable in high levels in cervicovaginal discharges. In the normal pregnancy, cervical glands secrete HCG into cervicovaginal secretions, but its concentration is less than 10 mIU/mL after 20th gestational week.[15] Thus, the cutoff value equal to 25 mIU/mL that was used in our study is appropriate.

In this study, similar to other studies, the gold standard for diagnosis of PROM was three tests including direct speculum examination, fern test, and nitrazine test.[10][12][13][16] However, inclusion criteria such as interfering factors of these tests could be controlled; therefore, the patients were excluded from study in the presence of signs or symptoms of vaginitis, cervisitis, using vaginal medications or having intercourse in the prior night, visible blood in vaginal fluid and presence of meconium in amniotic fluid. In Cooper et al.'s study, only vaginal bleeding was the exclusion criteria and patients with vaginitis underwent treatment prior to study.[8] It seems that applying more exclusion criteria in our study resulted in significantly lower false positive results in fern and nitrazine tests in comparison to that study.

Diagnostic parameters of ELISA for HCG in Shahin et al.'s study (2007) were 72%, 84%, 75%, 81.5%, and 78%, respectively.[17] Kariman et al. utilized cutoff 22.3 mIU/mL for ßHCG and found that its sensitivity for diagnosis of PROM was 95.3% and specificity, PPV, NPV and accuracy were 97.7%, 97.6%, 95.5%, and 96.5%, respectively.[15] Kim et al. (2005) used a higher cutoff value equal to 39.8 mIU/mL in their study and reported a sensitivity, specificity, PPV, and NPV of 95.5%, 94.7%, 91.3%, and 97.3%, respectively.[9] The sensitivity, specificity, positive and negative predictive values for ßhCG washing fluid in Taheripanah et al.'s study (2009) were 99.33%, 69.85%, 69.4% and 69.6%, respectively.[18]

Several studies have demonstrated that cervicovaginal ßhCG in pregnancies complicated with PROM is significantly higher than normal pregnancies. Anai et al. study (1997) is the only study that cervicovaginal ßhCG was measured in all trimesters of pregnancy and its values in first, second and third trimesters were 6.3, 9.5, and 37.9 mIU/mL, respectively.[14] In their study, utilizing the cutoff 50 mIU/mL for cervicovaginal ßhCG in third trimester resulted in sensitivity of 100%, specificity 96.5%, PPV 88.9%, NPV 100%, and accuracy 97.2%. The only study which demonstrated that cervicovaginal ßhCG levels reach to a relatively stable level after the first trimester of pregnancy. The high cutoff value in Esim et al. study may be due to participation of women in all trimesters of pregnancy.

Based on our results, Cooper[8] and Kariman[16] studies, it seems logical to say that one step cervicovaginal ßhCG dipstick is a reliable, easy, cheap and in available diagnostic test for PROM. Other advantage of this method is that only contamination of specimen with blood may affect the true results of this method, while the fern or nitrazine tests may be affected by many factors. These dipsticks are easily and rapidly available in all settings without any need to complex laboratory equipments.

Our results and above mentioned studies revealed that quantitative assay of HCG by ELISA for diagnosis of PROM was more reliable than usual methods such as fern or nitrazine tests and even speculum examination as measurement of HCG are not affected by interfering factors like fern or nitrazine test. On the other hand, when speculum examination is performed with a delay after rupture of membranes, it may not be helpful, while, high concentration of HCG can be detectable in cervicovaginal secretions at least 24 hours after rupture of membranes.[12]

Finally, this study showed that HCG measurement in cervicovaginal discharge with ELISA had an acceptable diagnostic value as high as ßhCG. While, HCG assay kits are cheaper than ßhCG. Moreover, HCG kits for detection of chorionic gonadotropins and thereby pregnancy is presently being produced in Iran.
